# Argatroban versus Lepirudin in critically ill patients (ALicia): a randomized controlled trial

**DOI:** 10.1186/s13054-014-0588-8

**Published:** 2014-10-25

**Authors:** Tanja A Treschan, Maximilian S Schaefer, Johann Geib, Astrid Bahlmann, Tobias Brezina, Patrick Werner, Elisabeth Golla, Andreas Greinacher, Benedikt Pannen, Detlef Kindgen-Milles, Peter Kienbaum, Martin Beiderlinden

**Affiliations:** Department of Anaesthesiology, Duesseldorf University Hospital, Heinrich-Heine University Duesseldorf, Moorenstrasse 5, 40225 Duesseldorf, Germany; Institut für Immunologie und Transfusionsmedizin, Ernst-Moritz-Arndt Universität Greifswald, Sauerbruchstrasse, 17489 Greifswald, Germany; Department of Anaesthesiology, Marienhospital Osnabrück, Bischofsstraße 1, 49074 Osnabrueck, Germany

## Abstract

**Introduction:**

Critically ill patients often require renal replacement therapy accompanied by thrombocytopenia. Thrombocytopenia during heparin anticoagulation may be due to heparin-induced thrombocytopenia with need for alternative anticoagulation. Therefore, we compared argatroban and lepirudin in critically ill surgical patients.

**Methods:**

Following institutional review board approval and written informed consent, critically ill surgical patients more than or equal to 18 years with suspected heparin-induced thrombocytopenia, were randomly assigned to receive double-blind argatroban or lepirudin anticoagulation targeting an activated Partial Thromboplastin Time (aPTT) of 1.5 to 2 times baseline. In patients requiring continuous renal replacement therapy we compared the life-time of hemodialysis filters. We evaluated in all patients the incidence of bleeding and thrombembolic events.

**Results:**

We identified 66 patients with suspected heparin-induced thrombocytopenia, including 28 requiring renal replacement therapy. Mean filter lifetimes did not differ between groups (argatroban 32 ± 25 hours (*n* = 12) versus lepirudin 27 ± 21 hours (*n* = 16), mean difference 5 hours, 95% CI −13 to 23, *P* = 0.227). Among all 66 patients, relevant bleeding occurred in four argatroban- versus eleven lepirudin-patients (OR 3.9, 95% CI 1.1 to 14.0, *P* = 0.040). In the argatroban-group, three thromboembolic events occurred compared to two in the lepirudin group (OR 0.7, 95% CI 0.1 to 4.4, *P* = 0.639). The incidence of confirmed heparin-induced thrombocytopenia was 23% (*n* = 15) in our study population.

**Conclusions:**

This first randomized controlled double-blind trial comparing two direct thrombin inhibitors showed comparable effectiveness for renal replacement therapy, but suggests fewer bleeds in surgical patients with argatroban anticoagulation.

**Trial registration:**

Clinical Trials.gov NCT00798525. Registered 25 November 2008

**Electronic supplementary material:**

The online version of this article (doi:10.1186/s13054-014-0588-8) contains supplementary material, which is available to authorized users.

## Introduction

Heparin is the standard anticoagulant in critically ill patients [1]. However, anticoagulation remains a challenge because these patients have an increased risk for thromboembolism and for bleeding [2], often suffer from multiorgan impairment and are often thrombocytopenic. Despite multiple causes for thrombocytopenia, heparin-induced thrombocytopenia (HIT) warrants special attention. HIT is a pro-thrombotic syndrome caused by antibodies against complexes of platelet factor 4 and heparin, which activate platelets, causing platelet aggregation and hypercoagulability [3]. Thus, in HIT the risk of thrombosis increases paradoxically during heparin administration. The overall incidence of confirmed HIT in critically ill patients in general is relatively low, in the range of 0.5% [4], but increases up to 28% in subgroups preselected by clinical symptoms [5]. As soon as HIT is suspected, patients require an alternative anticoagulant [6]. At the time of this study, two approved direct thrombin inhibitors, argatroban and lepirudin, were available for alternative anticoagulation in HIT patients, but no prospective comparative studies between these drugs had been performed to date. In contrast to lepirudin, argatroban might be beneficial due to its hepatic elimination, short half-life and thrombolytic activity, particularly in patients with acute kidney injury [7-9]. In critically ill patients disposed to developing HIT and the need for renal replacement therapy, filter clotting is a major complication, which also needs to be prevented by appropriate alternative anticoagulation [10].

In this prospective randomized study we compared argatroban and lepirudin to evaluate their efficacy and safety in critically ill surgical patients with a special focus on filter life time during continuous renal replacement therapy.

## Materials and methods

The Ethics Committee of the Medical Faculty, Heinrich-Heine-University Düsseldorf, Germany and the Bundesinstitut für Arzneimitel, EudraCT number 2006-003122-28 approved this study (ClinicalTrials.gov number NCT00798525. Registered 25 November 2008), which has been performed in accordance with the Declaration of Helsinki in its effective form. Written informed consent was obtained from patients or their legal guardian. In this double blind trial we included surgical intensive care unit patients with expected ICU treatment >24 hours, age ≥18 years and suspected HIT (decrease in platelet count >50% from baseline, persisting for more than 24 hours, 4 T-Score >3 [11,12] or positive PF4/heparin enzyme-linked immunosorbent assay). Exclusion criteria were: active bleeding, intracranial surgery, spontaneous activated partial thromoplastin time (aPTT) >60 seconds, known HIT (treated with open label argatroban), adverse events against study drugs and pregnancy.

### Primary endpoint

The primary endpoint was a mean life-time of a maximum of two consecutive filters in patients with continuous renal replacement therapy.

Secondary endpoints (all patients) were: 1) relevant bleeding [13]: moderate (transfusion required) or severe (intracranial or hemodynamic compromise requiring intervention); 2) transfusion requirements; 3) objectively confirmed new thromboembolism; 4) anaphylactoid reactions; 5) duration of intensive care unit and hospital stay; 6) in-hospital mortality; and 7) hours until first aPTT 55 to 65 seconds.

### Study protocol

In case of suspected HIT, heparin was stopped and patients were randomized to argatroban (Mitsubishi Pharma Europe, London, UK) or lepirudin (Celgene, Munich, Germany), adjusted to an aPTT of 55 to 65 seconds (1.5 to 2 times baseline (<37 seconds); Pathromtin SL, Siemens Healthcare Diagnostic Products GmbH, Marburg, Germany). Computer generated multi-block 1:1 randomizations were kept in sealed opaque envelopes. Results from external heparin-induced platelet activation assay (HIPA) are usually available within approximately seven days. Therefore, per protocol data collection was limited to a maximum of seven days. In the case of a negative result of HIPA testing, heparin anticoagulation was started again. In case of a positive HIPA test, alternative anticoagulation was continued as long as considered necessary to treat or prevent thrombembolic complications.

### Preparation and dosing of study drugs

Study drugs were prepared by personnel not involved in data collection and delivered to the intensive care unit in neutral 50 ml syringes. The personnel preparing the study drug were informed on the renal and hepatic function of each patient and filled the syringes accordingly with different drug concentrations ensuring that the treating physician always started with identical infusion rates of 0.05 ml/kg/hour at initiation of treatment.

Lepirudin: 1) patients with continuous renal replacement therapy: final concentration 0.1 mg/ml, initiated as a continuous infusion of 5 μg/kg/hour; 2) patients with moderate renal impairment (creatinine ≥1.3 mg/dl): final concentration 0.2 mg/ml, initial infusion of 10 μg/kg/hour; 3) patients without renal impairment (creatinine <1.3 mg/dl): final concentration 1 mg/ml, initial infusion 50 μg/kg/hour.

Argatroban: 1) patients without liver dysfunction: final concentration 0.6 mg/ml, initiated as continuous infusion of 0.5 μg/kg/minute; 2) patients with severe liver dysfunction (bilirubin of >4 mg/dl): final concentration 0.3 mg/ml, initial infusion 0.25 μg/kg/minute. Study drug infusion rates <0.1 ml/hour were assessed as a stop of infusion.

### Renal replacement therapy

Renal replacement therapy was initiated in the case of: 1) fluid overload resistant to diuretic therapy (maximum dose of furosemide 1,000 mg/day); 2) severe metabolic (renal) acidosis, pH <7.2; 3) hyperkalemia >6.5 mmol; 4) uremic symptoms (pericarditis, encephalopathy); 5) elevated serum urea (>150 mg/day) or creatinine (>3.0 mg/dl); or 6) glomerular filtration rate <15 ml/minute. We performed continuous veno-venous hemodiafiltration via Niagara venous catheters (13.5 french, 2 lumen, 20 cm, C.R. Bard GmbH, Karlsruhe, Germany) using a saline primed dialysis circuit with post dilution. Blood flow was adjusted to 120 to 150 ml/hour. Ultrafiltration rate depended on the individual goal of the daily net fluid balance per patient (0 to 300 ml/hour) using Ultraflux AV1000 filter (Fresenius Medical Care, Bad Homburg, Germany). We used a dialysate to ultrafiltration flow ratio of 1:1, resulting in a filtration fraction of about 27%. Anticoagulation was adjusted to target aPTT prior to the start of dialysis. In case of circuit clotting, a second system was initiated. In case of repeated clotting, the study endpoint was reached and regional citrate anticoagulation added, but the life-times of these filters were not analyzed.

### Measurements

For aPTT control, blood samples were taken two hours after study drug initiation, every four hours until target aPTT was reached and then every eight hours or as deemed necessary by the treating physician. Routine blood samples were taken at least every morning, according to institutional routine. On-site screening for HIT was performed using an enzyme-linked immunosorbent assay for PF4/heparin (until December 2011: Haemochrom Diagnostica GmbH, Essen, Germany since then Technoclone GmbH, Vienna, Austria) with a cut-off of 0.4 optical density units. Sera of the study patients were further tested by HIPA [14].

Control of study drug application: duration, infusion rates and interruptions in application were documented. Infusion rates of less than 0.1 ml/hour were assessed as a stop of study drug infusion.

### Statistics

Based on pilot data, a prolongation of mean hemodialysis circuit life-time of 30 hours for lepirudin to 57 hours with argatroban was considered to be clinically relevant (standard deviation 25 hours). A sample size of 15 renal replacement patients per group (80% power, two-sided alpha 0.05) required enrolment of 100 suspected HIT patients.

To allow comparison of coagulation variables at specific time points, values between two measurements were linearly extrapolated.

Using IBM SPSS Statistics 21, we performed an intention to treat analysis and used t-test or U-test and Chi-square or Fisher’s exact-test as appropriate with a two-sided level of significance <0.05. For analysis over time, Bonferroni corrections were applied. Kaplan-Meier estimator with log rank test was used to analyze mean filter life-time between the two groups.

## Results

**Figure 1:**
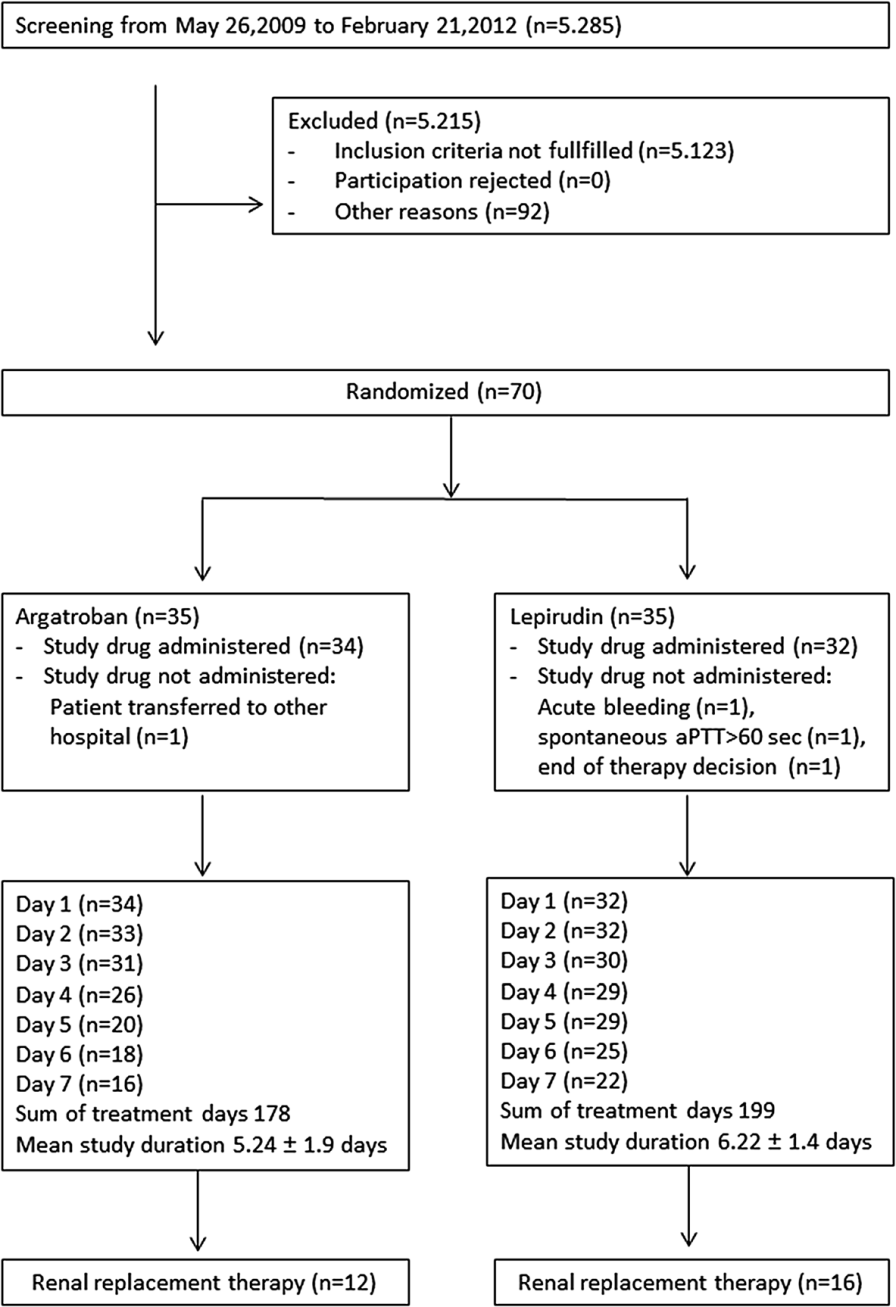
**Trial flow chart.** Data are presented as mean ± SD, n = number of patients, sec = seconds. SD, standard deviation.

Study drugs were administered to 66 patients (Trial profile, Figure 1), of whom 28 (42%) underwent continuous renal replacement therapy. Initial patient characteristics did not differ between the groups (Table 1). The study was terminated by 31 March 2012, when delivery of lepirudin was stopped by the manufacturer [15].

**Table 1:** **Patient characteristics prior to the start of the study drug**

	**Argatroban (number = 34)**	**Lepirudin (number = 32)**
Age (years)	68 ± 12	64 ± 17
Male (number)	24 (71%)	19 (59%)
Body mass index (kg/m^2^)	29 ± 9	26 ± 5
Simplified Acute Physiology Score II	35 ± 14	36 ± 14
SOFA score	10 ± 6	10 ± 4
Thromboembolic events (number)	11 (32%)	12 (38%)
Hemoglobin (g/dl)	10.1 ± 1.2	9.7 ± 1.0
Thrombocytes (per nl)	144 ± 124	152 ± 160
INR	1.2 ± 0.2	1.2 ± 0.3
aPTT (seconds)	46.1 ± 8.6	43.9 ± 7.9
Thrombin time (seconds)	27.7. ±29.2	19.0 ± 5.3
Duration of previous heparin therapy (days)	16 ± 4	14 ± 12
Probability of HIT according to 4 T Score (number)	0/22/12^a^	3/23/6^a^
(low /intermediate/high)	(0/65/35%)	(9/72/19%)
General and visceral surgery (number)	10	7
Vascular surgery (number)	4	2
Cardiac surgery (number)	19	18
Others (number)	1	5
Chronic renal insufficiency (number)	6	7

^a^4 T Scores were obtained for one patient in the argatroban and for two patients in the lepirudin-group with missing information and might therefore be underestimated. Data are presented as mean ± SD or full numbers and percentage in parenthesis. There were no statistically significant differences between the groups. aPTT, activated partical thromboplastin time; HIT, heparin-inducd thrombocytopenia; SD, standard deviation; SOFA = Sequential Organ Failure Assessment; INR, international normalized issue.

### Primary endpoint

**Figure 2:**
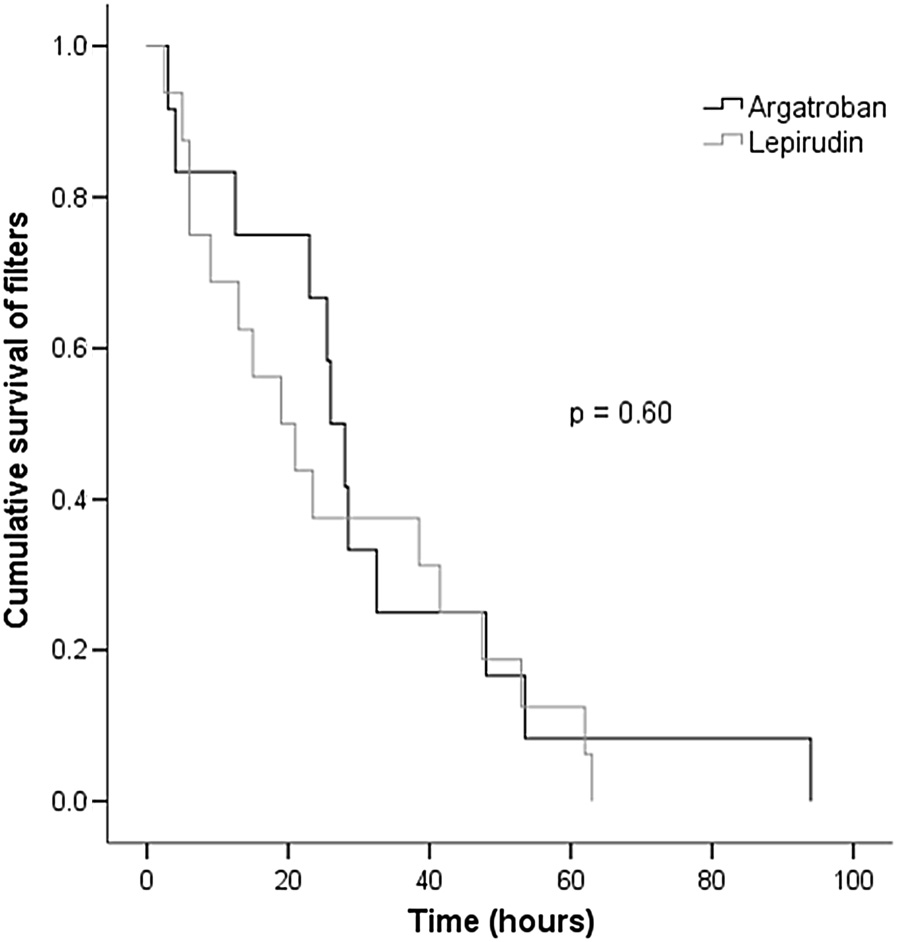
**Kaplan-Meier analyses of time till filter clotting.**

Mean life-time of a maximum of two consecutive dialysis circuits did not differ significantly between the two groups (Table 2 and Figure 2). Censoring filters which were ended electively due to patient transfer, operation or diagnostic testing did not alter our findings (data not shown). Mean dialysis dose was 36 ± 18 ml/kg/hour and did not differ significantly between groups, with a mean ultrafiltration rate of 110 ml/hour.

**Table 2:** **Results of the primary endpoint of the trial comparing the filter life-time between groups**

	**Argatroban (number = 34)**	**Lepirudin (number = 32)**	***P-value***	**95% Confidence interval**
Continuous renal replacement therapy	12 (35%)	16 (50%)	0.227	
Patients with two consecutive filters	9 (26%)	12 (37%)	0.424	
Life-time of first filter (hours)	33 ± 33	22 ± 22	0.298	−10 – 33
Life-time of second filter (hours)	29 ± 12	34 ± 34	0.681	−30 – 20
Average filter life time (hours)	32 ± 25	27 ± 21	0.574	−13 – 23

Data are presented as mean ± SD or full numbers and percentage in parenthesis. A *P*-value of <0.05 was considered to be statistically significant. SD, standard deviation.

### Secondary endpoints

Secondary endpoints are summarized in Table 3. Significant differences were found for the incidence of bleeding complications and the time till target aPTT.

**Table 3:** **Comparison of secondary endpoints and study drug application between the argatroban- and lepirudin-group**

	**Argatroban (number = 34)**	**Lepirudin (number = 32)**	***P***
Secondary endpoints			
Bleeding (number)	4 (12%)	11 (34%)	0.040
Moderate (number)	2 (6%)	8 (25%)	0.041
Severe (number)	2 (6%)	3 (9%)	0.667
Packed red blood cells (Units)	3 ± 8	4 ± 5	0.488
Fresh frozen plasma (Units)	3 ± 12	2 ± 5	0.582
Platelet transfusion (Units)	1 ± 4	1 ± 2	0.949
Newly diagnosed thrombembolic events (number)	3 (9%)	2 (6%)	0.639
Anaphylactoid reactions (number)	1(3%)	2 (6%)	0.608^a^
Length of ICU stay (days)	24 ± 22	32 ± 32	0.213
Length of hospital stay (days)	54 ± 41	53 ± 55	0.919
In-hospital mortality (number)	6 (18%)	9 (28%)	0.389
Time until target aPTT of 55 to 65 seconds (hours)	20 ± 19	11 ± 9	0.017
Time until aPTT ≥55 seconds (hours)	12 ± 16	8 ± 6	0.189
Patients with first aPTT >65 seconds (number)	9 (26%)	4 (12%)	0.213
First aPTT >65 seconds (seconds)	69 ± 3	74 ± 10	0.389
Study drug application			
Duration of study drug application (hours)	103 ± 47	126 ± 37	0.030
Infusion rate (ml/hour)	4.1 ± 2.9	3.8 ± 2.5	0.654
Interruption in study drug application (number)	12 (35%)	14 (43%)	0.615
Cumulative duration of interruptions (hours)	33 ± 39	34 ± 39	0.860

^a^None of the anaphylactoid reactions was attributed to the study drugs. Data are presented as mean ± SD or full numbers and percentage in parenthesis. A *P*-value of <0.05 was statistically significant. Further details on secondary endpoints are explained in the text.

In the lepirudin group, bleeding complications occurred in eleven patients which was a significantly higher rate compared to only four patients with argatroban (odds ratio (OR) 3.9, 95% confidence interval (CI) 1.1 to 14.0, *P* = 0.040). Of note, eight of these eleven patients in the lepirudin group suffered from diffuse bleeding from drains, wound site, catheters, skin and upper airway mucosa. In three patients, distinct bleeding sites were identified, that is, intrathoracic, intraabdominal (hepatic artery following pancreatic surgery) and lower gastro-intestinal tract. In one patient the actual bleeding source was not identified although hemoglobin dropped to 6.5 mg/dl without any explanation and transfusion was necessary. The onset of bleeding occurred on day two in seven cases, on day four in two cases and on day five in two cases.

In the argatroban group, we observed gastrointestinal bleeding in three patients. A fourth patient suffered from hepatic insufficiency following extended liver resection, requiring massive transfusion and surgical revision. The onset of bleeding in argatroban patients occurred on days 2, 4, 6 and 7.

In the lepirudin group, two patients developed new thromboembolic events: one had a cerebral and the second myocardial infarction. In the argatroban group, three patients had new thromboembolic events: one had cerebral infarction, another pulmonary embolism and the third suffered from myocardial infarction and gastrointestinal ischemia (OR 0.7, 95% CI 0.1 to 4.4, *P* = 0.639).

### Comparison of patients with and without relevant bleedings

There was no significant difference in aPTT values over time between patients with and without bleeding (see Additional file 1). Patients with bleeding were more likely to suffer from chronic renal insufficiency (67% versus 6%, *P* <0 001), to require renal replacement therapy (73% versus 41%, *P* <0.001) and had a higher mortality rate than those without (60% versus 12%, *P* <0.001). A detailed comparison is included in Additional file 2.

### HIT

Of all study patients, 15 (23%) were HIT positive with at least an intermediate 4 T score and a positive HIPA test, 10 (29%) in the argatroban and 5 (16%) in the lepirudin group (*P* = 0.240). The need for renal replacement therapy did not differ between HIT and non-HIT patients (60% versus 38%, *P* = 0.150) and mean filter life-time was also not different (26 ± 19 hours versus 31 ± 24 hours, *P* = 0.606). There were no statistically significant differences in study drug dosing between HIT and non-HIT patients. In HIT positive patients, three newly diagnosed thromboembolic events (3/10, 30%) were detected in the argatroban group as compared to none out of five in the lepirudin group (*P* = 0.505).

### Control of study drug application

The duration of study drug administration was significantly shorter in the argatroban group (Table 3). There were no significant differences in aPTT or infusion rates over time (data not shown). Mean doses of study drugs applied during the trial were as follows: lepirudin: 1) patients with continuous renal replacement therapy: 6 ± 4 μg/kg/hour (n = 17), 2) patients with moderate renal impairment (creatinine ≥1.3 mg/dl, n = 7): 9 ± 5 μg/kg/hour and 3) patients without renal impairment (creatinine <1.3 mg/dl, n = 8): 43 ± 43 μg/kg/hour; argatroban: 1) patients with severe liver dysfunction (bilirubin ≥4 mg/dl, n = 3): 0.1 ± 0.1 μg/kg/minute and 2) other patients (n = 31) 0.5 ± 0.3 μg/kg/minute. Argatroban doses tended to be lower in patients on dialysis (0.33 ± 0.25 versus 0.55 ± 0.38 μg/kg/minute, *P* = 0.090).

## Discussion

This is the first published randomized controlled double-blind study comparing two direct-thrombin inhibitors in critically ill surgical patients requiring alternative anticoagulation following suspicion of HIT. The essential finding was that argatroban and lepirudin are both equally effective with regard to anticoagulation and filter patency, while argatroban appears to be associated with a significantly lower rate of clinically relevant bleeding complications. However, absolute filter life-times are comparably short, thus additional regional citrate anticoagulation could optimize filter patency in this specific patient population.

We had planned to study 30 patients with renal replacement. Despite the early termination of the trial, we were still able to study 28 of the 30 patients. The difference in filter life-time between argatroban and lepirudin of only five hours yielded only a small effect size of 0.2 and is of minor clinical relevance. Therefore, completion of the trial as initially planned would not have had a significant effect on our results. Argatroban and lepirudin demonstrate equally effective anticoagulation of critically ill surgical patients with continuous renal replacement therapy. However, filter life times were comparably low and if repeated clotting occurred, we added regional citrate anticoagulation, but did not quantify the resulting life-times. Regional citrate anticoagulation is suggested as first choice in the Kidney Disease: Improving Global Outcomes (KDIGO) guidelines for acute kidney injury [16]. Therefore, based on our results, adding regional citrate anticoagulation could further optimize the treatment of critically ill patients suspected for HIT and renal replacement therapy. It is important to note that regional citrate anticoagulation does not substitute for alternative systemic therapeutic anticoagulation in HIT patients. Patients with suspected or proven HIT require systemic anticoagulation in therapeutic doses due to the pro-thrombotic nature of the disease [17]. Our data show that in patients with continuous renal replacement therapy systemic anticoagulation might not be sufficient to facilitate long filter patency. Thus, addition of regional citrate anticoagulation should be considered.

Our data suggest more moderate bleeding complications in the lepirudin group, despite similar aPTT and platelet counts in both groups. Although the ecarin clotting time is considered to be more appropriate, aPTT is much more widely used in clinical practice and has become accepted to guide anticoagulation by direct thrombin inhibitors and thus we used it to titrate both study drugs [18].

The significantly shorter time until the target aPTT between 55 and 65 seconds was reached in the lepirudin group does not explain the higher bleeding incidence, because argatroban patients did not reach the target aPTT later due to under dosing but rather because of an initial ‘overshoot’ , defined as a first aPTT above 65 seconds. Moreover, this overshoot in anticoagulation was not associated with an increased incidence of bleeding.

Despite very cautious administration of anticoagulants, more patients who underwent renal replacement therapy or lepirudin treatment bled and had a higher mortality. Patients with renal failure have an increased risk of bleeding due to impaired platelet function, vessel wall abnormalities and changes in the coagulation cascade [19]. However, a detailed analysis of factors contributing to these findings is beyond the scope of this trial.

Our trial design was unusual, as not all patients with suspected HIT could be expected to reach the primary endpoint. We recruited patients at the time point when HIT was suspected, need for renal replacement therapy was not an inclusion criterion. Restricting inclusion to patients who were undergoing hemodialysis at the time point of HIT suspect would have made the trial nearly impossible to conduct in a single center. After intensive discussion with our IRB and our national regulatory board, it was advised to include all patients suspected of having HIT into the trial, because alternative anticoagulation is required in these cases. We restricted the measurement to a maximum of two filters, because repeated clotting is expensive and poses an unnecessary risk to patients due to transfusion of packed red cells. Thus, according to clinical routine, we added regional citrate anticoagulation after clotting of the second filter.

As lepirudin is not marketed anymore, our observations have two major clinical implications with regard to argatroban.

First, a target aPTT of 1.5 to 2 times baseline, which we had chosen for both drugs based on our previous experience [20,21], is adequate in critically ill surgical patients with suspected HIT. The argatroban group in our study experienced a bleeding incidence of 12% (4/34) and thromboembolic complications in 8.8% (3/34). Doepker *et al*. found significantly higher incidences of bleedings, but similar thromboembolic complications in a mixed population of medical and surgical patients with argatroban anticoagulation adjusted to a baseline of 2 to 2.5 [22].

Second, patients with thrombocytopenia, persisting for at least 24 hours postoperatively, a 4 T score >3 indicating at least an intermediate risk for HIT or a positive PF4/heparin immunoglobulin G (IgG) ELISA, should receive alternative anticoagulation, despite other potential causes for thrombocytopenia. Using these criteria, we found as many as 23% of our patients to suffer from HIT, defined as a positive HIPA test.

Current guidelines recommend the 4 T score for suspicion of HIT and for the decision about alternative anticoagulation [23]. Consistent with a recent meta-analysis, the positive predictive value of the 4 T score for HIT was moderate and the majority of patients with confirmed HIT had only an intermediate risk score [12]. Additionally, the positive predictive value of positive PF4/heparin IgG ELISA was also low. These tests often detect antibodies, which may not necessarily be the cause of the clinical symptoms of HIT [24,25]. Therefore, even with careful application of the 4 T score and HIT ELISA testing, a subsequent platelet activation assay is indispensable to prove or rule out HIT in critically ill patients.

### Limitations of the trial

This is a single-center trial, conducted pragmatically close to clinical routine in a limited number of critically ill surgical patients. Therefore, our data may not be used to generalize the effects on other patient populations. The early termination of the trial is, however, a major limitation. Nevertheless, data on the safe and appropriate application of argatroban are important for the treatment of surgical patients because this population is at highest risk for bleeding. With regard to the comparable aPTTs in both groups, we consider the dosing regimens clinically appropriate for critically ill surgical patients.

The comparably low filter life-times measured in this trial might not only relate to the systemic anticoagulation, but are also influenced by other factors, such as filter area, blood flow and filtration fraction. Thus, we are not promoting our renal replacement strategy but provide a comparison of argatroban and lepirudin under the same premises. Further research is needed to address ways to improve filter patency in patients with HIT requiring continuous renal replacement therapy.

## Conclusions

Argatroban and lepirudin provide equally effective anticoagulation in critically ill surgical patients requiring continuous renal replacement therapy. In patients receiving lepirudin compared to argatroban, we observed a significantly higher incidence of clinically relevant bleeding complications. Accordingly, the results of our trial support the use of argatroban in patients with clinically suspected or confirmed HIT, who require continuous intravenous anticoagulation.

## Key messages

- In critically patients, suspected of heparin-induced thrombocytopenia undergoing continuous renal replacement therapy, filter-life time is comparable between argatroban and lepirudin.
- Argatroban is associated with a lower risk of bleeding in critically ill patients.

## Additional files

Additional file 1:
**Comparison of aPTT over time between patients with and without relevant bleedings.** Data are presented as mean ± SD. There was no significant difference. Additional file 2:
**Comparison of patients with and without bleeding.**

## Abbreviations

aPTT: activated partial thromboplastin time
CI: confidence interval
ELISA: enzyme-linked immunosorbent assay
HIPA: heparin-induced platelet activation assay
HIT: heparin-induced thrombocytopenia
OR: odds ratio
