# Supplementary figures and images for: Argatroban versus Lepirudin in critically ill patients (ALicia): a randomized controlled trial
Source: Crit Care. 2014 Oct 25;18(5):588. doi: 10.1186/s13054-014-0588-8 (PMC4234853; doi:10.1186/s13054-014-0588-8)

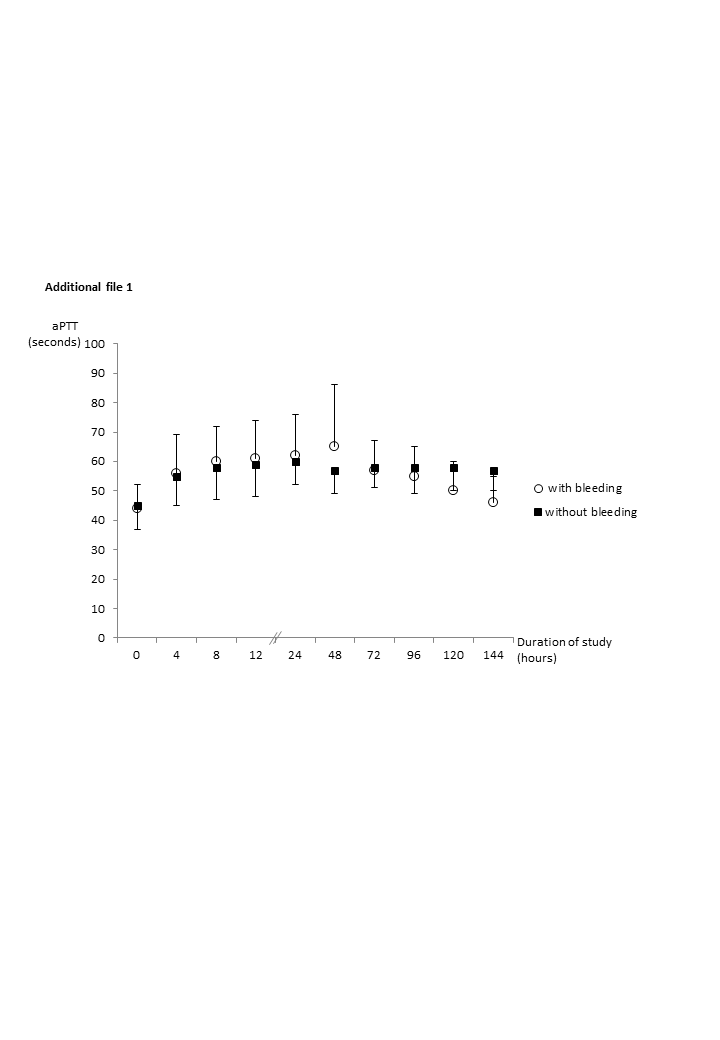

Supplement: Additional file 1: — Comparison of aPTT over time between patients with and without relevant bleedings. Data are presented as mean ± SD. There was no significant difference. [file 13054_2014_588_MOESM1_ESM.tiff]
