# Supplementary material for: Argatroban versus Lepirudin in critically ill patients (ALicia): a randomized controlled trial
Source: Crit Care. 2014 Oct 25;18(5):588. doi: 10.1186/s13054-014-0588-8 (PMC4234853; doi:10.1186/s13054-014-0588-8)
Supplement: Additional file 2: — Comparison of patients with and without bleeding. [file 13054_2014_588_MOESM2_ESM.doc]

Additional file 2. Comparison of patients with and without bleeding

|  | Pat. with bleeding  (n=15) | Pat. without bleeding  (n=51) | *P* |
| --- | --- | --- | --- |
| Age (years) | 60 ± 17 | 68 ± 14 | 0.061 |
| Male (n) | 9 (60%) | 34 (67%) | 0.760 |
| Body mass index (kg/m^2^) | 27 ± 8 | 28 ± 7 | 0.600 |
| Simplified acute physiology score II | 40 ± 12 | 34 ± 14 | 0.095 |
| Sequential organ failure assessment score | 11 ± 4 | 9 ± 5 | 0.453 |
| Patients with thrombembolic events at inclusion (n) | 2 (13%) | 20 (39%) | 0.071 |
| Chronic renal insufficiency (n) | 10 (67%) | 3 (6%) | <0.001 |
| Hemoglobin (g/dl) | 9. 5 ± 1.0 | 10.0 ± 1.1 | 0.104 |
| Platelets (per nl) | 163 ± 170 | 144 ± 134 | 0.639 |
| INR | 1.2 ± 0.3 | 1.2 ± 0.2 | 0.513 |
| aPTT (sec) | 44 ± 8 | 45 ± 8 | 0.530 |
| Thrombin time (sec) | 27 ± 24 | 22 ± 21 | 0.419 |
| Duration of previous heparin therapy (days) | 16 ± 4 | 15 ± 17 | 0.799 |
| Probability of HIT according to 4 T Score  (low /intermediate/high) (n) | 2/9/4  (13/60/27%) | 1/36/14  (2/71/27%) | 0.259 |
| Lepirudin treatment (n) | 11 (73%) | 21 (41%) | 0.040 |
| Positive HIPA test (n) | 3 (20%) | 12 (24%) | 0.747 |
| General and visceral surgery (n)  Vascular surgery (n)  Cardiac surgery (n)  Others (n) | 6 (40%)  1 (7%)  6 (40%)  2 (13%) | 10 (20%)  5 (10%)  31 (61%)  5 (9%) | 0.186 |
| New Thrombembolic events during trial (n) | 1 (7%) | 4 (8%) | 0.880 |
| Renal replacement therapy during trial (n) | 11 (73%)^#^ | 17 (33%) | 0.006 |
| Duration of hospitalization (days) | 51 ± 23 | 54 ± 54 | 0.817 |
| Duration of hospitalization in survivors (days) | 59 ± 23 | 55 ± 57 | 0.867 |
| Duration of ICU stay (days) | 38 ± 23 | 25 ± 21 | 0.055 |
| Mortality (n) | 9 (60%) | 6 (12%) | <0.001 |

Data are presented as mean ± SD or full numbers and percentage in parenthesis. A p-value of <0.05 was statistically significant. n=number of patients, sec=seconds, ^#^of these 7 patients were in the lepirudin group
